# Sustained response to symmetry in extrastriate areas after stimulus offset: An EEG study

**DOI:** 10.1038/s41598-019-40580-z

**Published:** 2019-03-13

**Authors:** Marco Bertamini, Giulia Rampone, Jennifer Oulton, Semir Tatlidil, Alexis D. J. Makin

**Affiliations:** 1 University of Liverpool, Department of Psychological Science, Liverpool, L697ZA UK; 2 University of Liverpool, School of Psychology, Liverpool, L697ZA UK; 3 Liverpool John Moores University, Sport and Exercise Sciences, Liverpool, L2 2QP UK

## Abstract

Electrophysiological (EEG) studies of human perception have found that amplitude at posterior electrodes is more negative for symmetrical patterns compared to asymmetrical patterns. This negativity lasts for hundreds of milliseconds and it has been called *sustained posterior negativity* (SPN). Symmetry activates a network of visual areas, including the lateral occipital complex (LOC). The SPN is a response to presence of symmetry in the image. Given the sustained nature of this activation, in this study we tested the persistence of the SPN after stimulus offset. Two shapes were presented (for 0.5 s each) with a 1 s blank interval in between. We observed a sustained response after stimulus offset, irrespective of whether the task required processing of shape information. This supports the idea that the response to symmetry is generated by information in the image, independently of task, and that it is sustained over approximately one second post stimulus onset.

## Introduction

**Figure 1:**
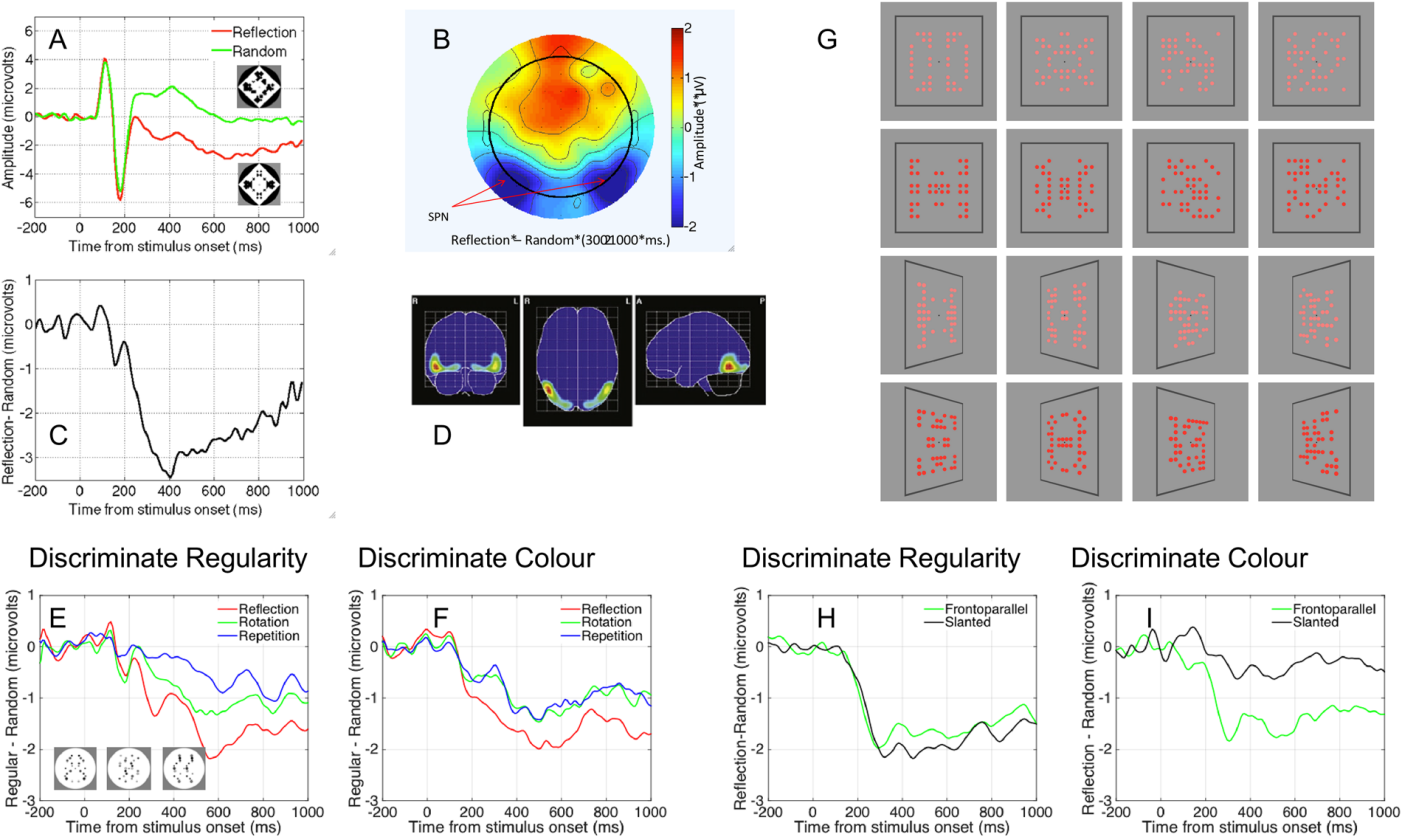
Panels A–D Results from Makin *et al*.^22^. (**A**) Grand Average ERP waves in reflection and random conditions (see inset for typical stimuli). (**B**) The SPN as a difference wave (reflection – random). (**C**) Topography of the SPN. (**D**) Anatomical locations of the SPN generators estimated with LORETA. Panels E and F show results from Makin *et al*. (Makin *et al*., 2013). The SPN for reflection, rotation and repetition were comparable whether participants were discriminating regularity or performing a oddball discrimination task. (**G**–**I**) shows results from Makin, Rampone and Bertamini^31^. The SPN was invariant when participants were discriminating regularity (**H**) but only responded to image symmetry when discriminating colour (**I**). Note that extraction of symmetry only occurred during active symmetry discrimination. All ERPs from PO7/8 electrodes (also used in current study).

Visual symmetry has an important role in the study of visual perception, and empirical work on perception of symmetry has a long history^1–6^. Neurophysiological and neuroimaging work has found evidence, using different methodologies, of tuning to image regularity within ventral, lateral and dorsal occipital cortex, including the Lateral Occipital Complex^7^. For example, several ERP studies have shown a regularity-related negativity over parieto-occipital areas (Fig. 1). In this paper, we briefly review this literature, and then present new data about the persistence of the symmetry response after stimulus offset. Observers compared patterns presented in two intervals (500 ms each) separated by a 1000 ms blank interval. This presentation is short compared to that used in previous EEG studies (typically more than 1 s). We use this procedure to (i) analyse persistent activity during the blank interval, and (ii) test whether this persistent activity is present also when shape information is not relevant for the task. In addition, we will analyse the response to the second pattern as a function of its relationship with the first pattern.

Observers compared the patterns (symmetrical or asymmetrical) and decided whether they were identical or different. Note that this is a task requiring processing of shape, but not necessarily processing of symmetry. We compared this shape matching task to a different task in which observers were not required to process the shape relationship between patterns (i.e. colour task). It is possible that when participants are engaged in matching the stimuli based on shape information, response to symmetry continues after the first offset; on the contrary when colour is attended, symmetry-related activity might be reduced or absent. To anticipate our results, we report that the activation continues after stimulus offset in both tasks, and this activation does not change (it is not enhanced) when the task requires a comparison and therefore a processing of shape information. With respect to the response to the second stimulus, symmetry-response was enhanced in the second interval when a novel symmetrical exemplar was shown, more than when the same symmetrical pattern was repeated, suggesting a form of symmetry priming.

The main focus of the study is the persistence of the response to a visual stimulus after offset, using electroencephalography (EEG) and the analysis of visual evoked potential (VEP). The International Society for Clinical Electrophysiology of Vision (ISCEV) defines three standard VEP protocols based on the type of stimuli used: (a) Pattern-reversal, (b) Pattern onset/offset and (c) Flash^8^. For pattern reversal there is no distinction between onset and offset, and the typical VEP has an initial negative deflection (N70), a prominent positive peak (P100), and a later negative component (N155). For pattern onset/offset, the time course is similar but with reversed polarity. Flash VEP are more variable, and they can include a slightly later positivity. Overall, what is known about VEP is that they produce clear signals within a short time window, and they do not include a sustained response.

Our hypothesis is that information about symmetry generates an activation that may be more long lasting that a typical VEP. We already know several aspects of the response to symmetry (reviewed below) but this is the first time we analyse activation after stimulus offset.

### Neurophysiological studies of visual symmetry

Behavioural studies have found that symmetry is detected quickly and efficiently^1,9–11^ and neuroimaging studies have identified neural areas active during symmetry perception in the human brain^7,12,13^. A series of fMRI studies have shown that the Lateral Occipital Complex (LOC) and other extrastriate areas (V4, VO1) are activated by symmetry, while primary visual areas are not^14–18^. Converging evidence comes from TMS studies, which have additionally found a causal role for the LOC^19,20^.

Jacobsen and Höfel^21^ analysed event related potentials (ERP) and found that amplitude at posterior electrodes was more negative for symmetrical than asymmetrical patterns. This difference wave is known as the *Sustained Posterior Negativity* (SPN, Fig. 1A–D). The SPN is generated by the extrastriate symmetry network^22^ and its amplitude scales with the salience of different symmetries^23–25^.

The SPN is stimulus-driven in the sense that it occurs independently of the participant’s task. For example, Makin, Rampone, Pecchinenda and Bertamini^24^ found similar SPN waves when participants were discriminating regularity type (Fig. 1E) or looking out for rare oddball trials (Fig. 1F). The rank order of SPN amplitudes for reflection, rotation and repetition was also task independent. The SPN is similar whether participants are making descriptive (symmetry/asymmetry) or evaluative (beautiful/ugly) judgements^26^. Likewise, Höfel and Jacobsen^27^ found that the SPN was similar when participants were actively discriminating regularity or passively viewing the patterns, while Höfel and Jacobsen^28^ found that the SPN was unchanged when participants deliberately misreported their answers. Makin, Rampone, Wright, Martinovic and Bertamini^29^ found that the SPN was similar when participants were discriminating regularity or number of objects. Although the SPN can be reduced when participants are reading superimposed negative words, it is unaffected by positive words^30^. Other work has shown that the SPN can be recorded when participants are attending to the colour of the stimulus^31,32^.

In steady-state visual evoke potential (SSVEP) paradigms with periodically alternating regular and irregular patterns, the extrastriate symmetry response can be isolated in the amplitude of odd harmonics. Like the SPN, the odd harmonic response happens even during passive viewing or when participants are engaging in secondary tasks^16,33–35^. Finally, fMRI studies have shown an extrastriate symmetry response when participants attended to colour rather than regularity^15^. Based on such evidence, recent review articles have concluded that the extrastriate symmetry response is task independent^7,12^.

So far, we have discussed experiments where symmetry was present in the image. Observers, however, can extract symmetry in other situations, for example in the case of symmetrical objects seen in perspective. Perspective may remove symmetry information in the 2D projection, depending on the relationship between axis of symmetry and axis of rotation, and regularity of the original shape is likely to decrease as the slant angle increases. The SPN response to *image symmetry* is task independent, as we have seen, but there is a response to *object symmetry* (symmetry not present in the image) when symmetry is task relevant. Makin *et al*.^31^ used configurations of small objects presented within a plane that was either frontoparallel or slanted by 50 deg. When participants were attending to the colour of the elements, amplitude was reduced by perspective (to the extent predicted by the specific transformation). However, amplitude was not altered when participants attended to symmetry. Therefore, the symmetry-sensitive network can go beyond information in the image and extract symmetry of an object when symmetry is relevant for the task.

Another situation where symmetry can be detected even though it is not present in the image is when partial information is made available over time. In a recent series of experiments Rampone *et al*.^36^ presented vertical reflections as two temporally separated halves. One half was presented for 500 ms, and when this was occluded the other half became immediately visible (e.g. left side then right side or vice versa). Therefore, there was no image symmetry on any individual frame; reflection could be only detected by the integration of the two images. When participants attended to the correspondence between the halves, the onset of the second half of the pattern generated an SPN^37^. This indicates that representations of object’s symmetry can be constructed by binding new information with older information stored in a perceptual buffer^38^. Here by object we mean the result of a process of grouping and perceptual organisation, in this case taking place over time. Importantly, when participants were attending to colour, this object symmetry response was absent.

In summary, both EEG and fMRI studies have shown activation in response to image symmetry^15,31^. Moreover, when symmetry is task relevant, the network also responds to object-level symmetry not present in the image. Symmetry is often a property of an object, and is classically described as a Gestalt grouping factor^39,40^. It should be noted that something similar to the SPN (a negative difference) has been reported for familiar objects versus abstract objects^41^, and for closed versus open shapes^42^. The extrastriate visual areas that are sensitive to symmetry are also sensitive to object information. However, the SPN is not simply a response to the presence of an object (objects are also present for asymmetrical stimuli), the SPN is present for closed polygons and for configurations of elements (e.g. dot patterns), and symmetry, unlike contour closure or familiarity, produces no N1 modulation^29,42^.

### Persistence of the SPN after stimulus offset

As we have seen converging evidence shows that visual symmetry is a powerful stimulus and the visual system is sensitive to the presentation of images containing symmetry even in abstract configurations. In the context of ERP studies, there is a characteristic response called sustained posterior negativity (SPN). In this paper we focus on this sustained aspect. In previous studies stimuli were presented for intervals of several hundred milliseconds, and the sustained response may therefore have been a result of long exposure. In this study we test whether this signal disappears when the image is removed, or whether the SPN, given that symmetry can be perceived with short presentations, will be present for some time after offset. The nature of SPN persistence will inform future models of symmetry processing.

### A study with multiple patterns and a matching task

To examine the fate of the SPN after the stimulus has disappeared we developed the following procedure. A pattern (symmetrical or asymmetrical) is presented for 500 ms, followed by a blank interval (1000 ms) and a second pattern (500 ms). We are interested in the activity within the retention interval, and how such activity depends on the task.

Based on the above review of the literature, there are three possible scenarios. (1) After stimulus offset there is no detectable activity related to the (previous) presence of symmetry, (2) The SPN lasts for several hundred milliseconds and is not immediately terminated by the removal of the pattern. This activation, including its sustained nature, is image-driven even though the image has been removed, and it will not be affected by the task. Once triggered the time course is fixed. (3) Alternatively, post stimulus activation is modulated by task. In other words, sustained activation is not always present. When shape is not task relevant, the SPN fades more rapidly after stimulus offset. Conversely, when shape is task relevant, the SPN persists across the retention interval.

**Figure 2:**
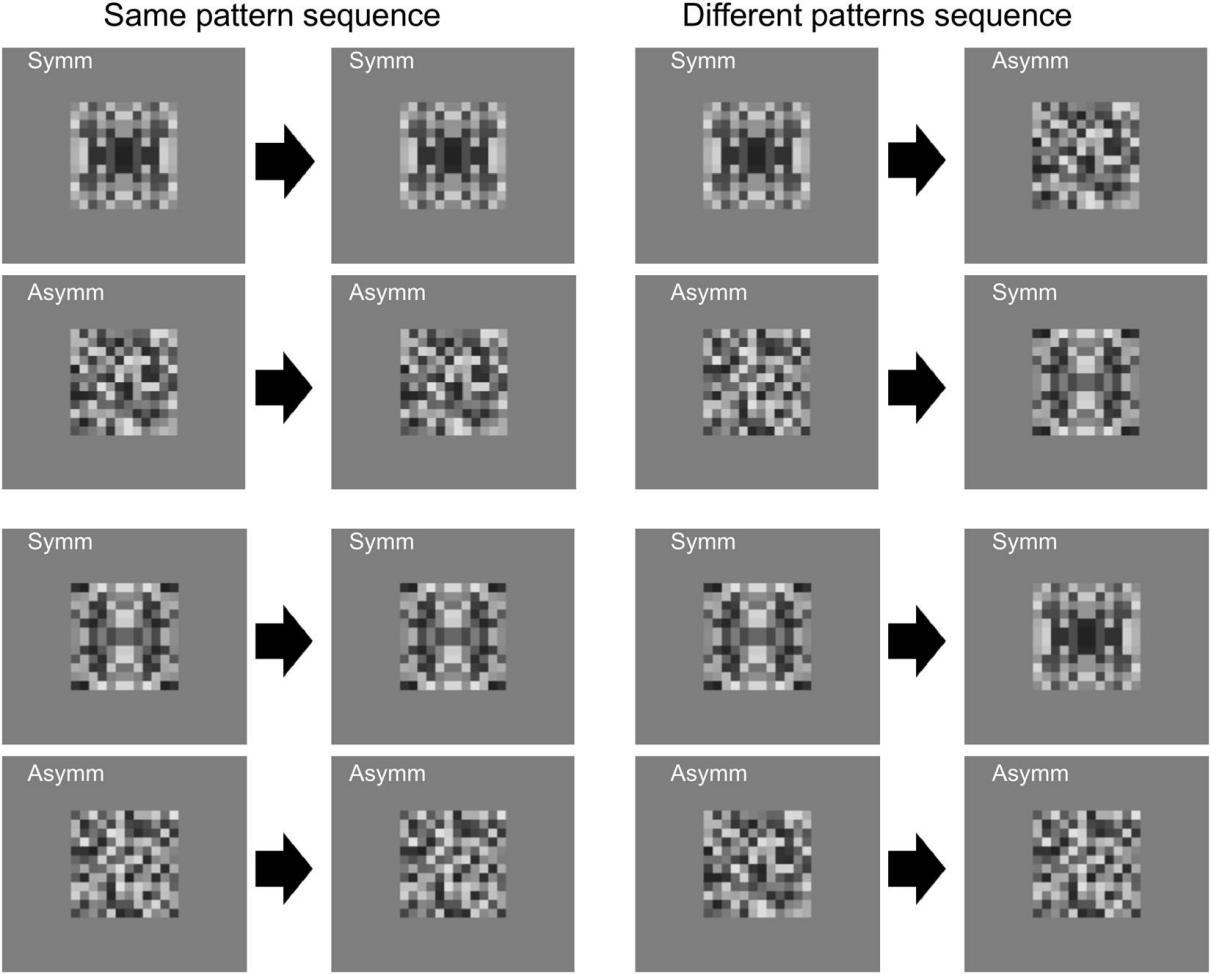
Examples of stimuli used in the experiment and their matches. In the shape matching task, participants judged whether the first and second pattern were the same (left panels) or different (right panels). In the Colour task, participants judged whether patterns were greyscale (as in these examples) or coloured (same pattern types but with blue colouration). Note that this figure represents the conditions presented in a block, hence the top and bottom panels of the left column duplicate each other.

**Figure 3:**
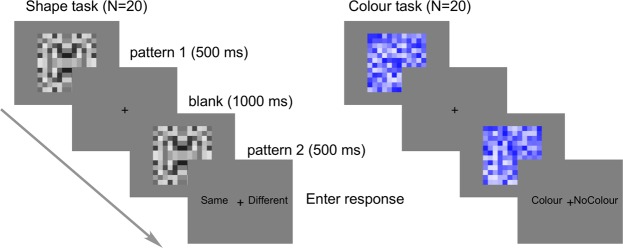
Trial structure in the Shape matching task (left) and Colour task (right). The examples show a Same trial with symmetrical patterns (left), and a Blue trial (right). In the Shape matching task, participants judged whether the first and second pattern were identical or different. In the Colour task, participants judged whether patterns were greyscale or blue.

To test these predictions, we compared the SPN in two tasks, shown in Figs 2 and 3. In the first interval an abstract pattern (symmetrical or asymmetrical) is presented. After a blank inter-stimulus interval, a second pattern appears. In the shape matching task participants judge whether the two patterns are identical. Therefore, the configuration of the first pattern has to be encoded and remembered. In the Colour task, there are some additional trials with a blue colouration, and participants perform a colour discrimination task. Therefore, in the Colour task pattern configuration is *not* relevant for the task.

Note that in neither task did the participants have to respond to symmetry per se. Previous results have shown that attention to symmetry is not necessary to generate a neural response to symmetry. However, only the shape matching task requires processing of the spatial configuration. The key comparison in this study is therefore novel and is a comparison between shape-relevant and non-shape-relevant tasks.

With this design, we can test the effect of repeated presentation of symmetry, and compare the response to the second pattern based not just on the task but on the relationship with the first pattern. Even when the second pattern is different from the first they could match in terms of symmetry (different exemplars). Therefore, it is possible to test whether a second response to symmetry is enhanced by a previous response to the same or a different exemplar.

It is known that responses to symmetry can be primed, and this is a factor we must consider. The evidence comes from behavioural studies. For patterns with multiple axes of reflection the salience of symmetry is higher when axes are orthogonal^43–45^. Starting from this observation, Treder, van der Vloed and van der Helm^46^ tested the interaction between symmetries. In a priming experiment they found priming when the prime and target had orthogonal axes. Priming, therefore, is not confined to the case of identical prime and target stimuli. In a recent paper, Sharman and Gheorghiu^47^ found that limiting the lifetime of the elements improves symmetry detection. They suggested an integration process in which signals are combined over time, as long as all elements are consistent with the same symmetry configuration.

There are three relevant time windows in our study, corresponding to the three events, and the analysis will be based on these windows excluding the first 200 ms: 200–500, 700–1500, and 1700–2000 ms. This analysis focuses on the SPN, excluding other early visually evoked components. The response to symmetry in the first pattern is expected to be similar in both tasks. We will then test to what extent the SPN persists in the Shape matching task and in the Colour task, and whether the task affects the response to the second pattern.

Finally, we will analyse the response to the second pattern in relation to the correspondence with the first pattern. Based on the literature on symmetry priming and spatial integration, we will test to what extent the amplitude is modulated by the repetition of an identical pattern or by the presentation of two patterns sharing the same axis of orientation.

## Results

### Behavioural results

Participants entered the correct judgement on most trials (94% correct on average in the Shape matching task, 99% correct rejections and 99% hits in the Colour task). This procedure therefore allows the encoding of shape information as well as colour information.

### ERP response to the first pattern

**Figure 4:**
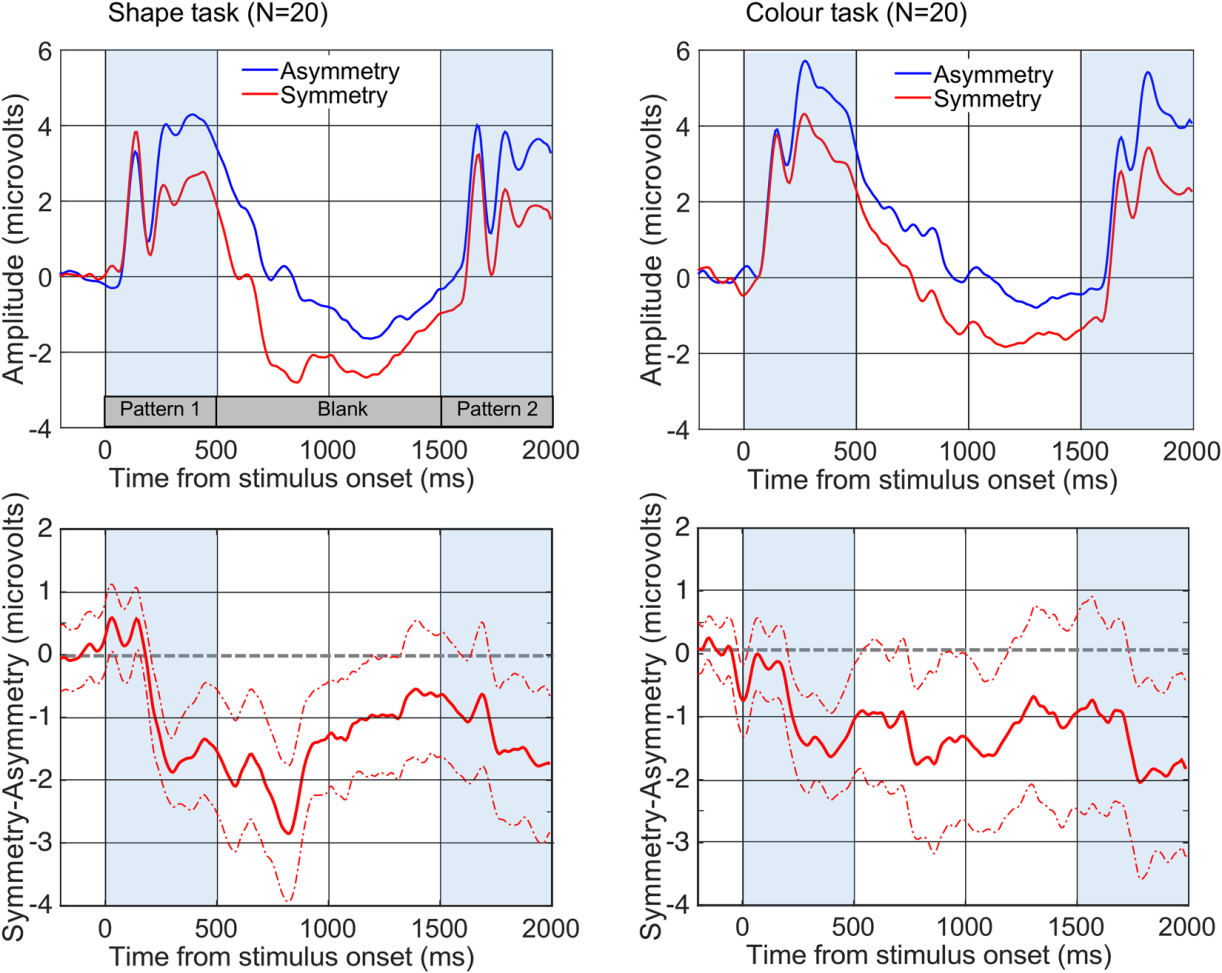
Grand Average ERPs. Left column shows results from the Shape matching task, the right column shows results from the Colour task. Top row: the asymmetry (blue) and symmetry (red) lines refer to the regularity of the first pattern, before the 1000 ms blank interval. These waves are averaged over Same and Different trials. Bottom row: SPN is shown as a difference wave (symmetry - asymmetry). Dotted lines indicate 95% CI.

Figure 4 shows that the initial, visually driven SPN at PO7/8 appeared around 200 ms after the onset of pattern 1, and the amplitude was similar in both tasks. A 2 × 2 mixed ANOVA [Regularity (Symmetry, Asymmetry) X Task (Shape, Colour)] found that amplitude in the 200–500 ms window was more negative for symmetrical than asymmetrical patterns (F (1, 38) = 43.534, p < 0.001, partial η^2^ = 0.534). There was no main effect of Task (F (1, 38) = 0.698, p = 0.409) and no Regularity X Task interaction (F (1, 38) = 0.128, p = 0.723).

### ERPs in the blank interval

Figure 4 shows that the SPN spanned the one-second blank interval in both tasks. To analyse this persistent SPN activity, we used the time window from 200 ms after stimulus offset until the end of the interval (700 to 1500 ms after the onset of pattern 1). There was again a main effect of Regularity (F (1, 38) = 12.341, p = 0.001, partial η^2^ = 0.245). There was no effect of Task (F (1, 38) = 2.232, p = 0.143) and no Regularity X Task interaction (F (1, 38) = 0.014, p = 0.906). The persistent SPN activity during the blank interval was statistically identical in both tasks.

### Mass Univariate analysis

**Figure 5:**
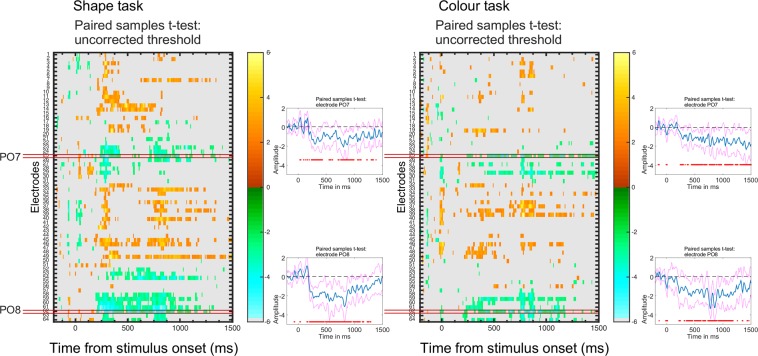
Mass univariate analysis. Colour scale shows t scores from paired t-test (Symmetry first vs. Random first, uncorrected for multiple comparisons). Negative t scores (symmetry < random) are blue/green, positive t scores (symmetry > random) are orange/red. All tests where p > 0.05 appear grey. X axis shows time from stimulus onset (including baseline, first pattern and blank) Y axis shows electrode number from the BioSemi 64 electrode montage. Electrodes PO7 and PO8 are highlighted (left). The SPN appears as a run of significant negative t values at these electrodes. The difference waves from PO7 and PO8 are shown in insets (95% confidence intervals in pink, signification difference from zero as red dot below).

Although ERP responses in the Shape and Colour task were similar, this analysis is spatiotemporally restricted. Are the tasks similar across all electrodes and time points, and are electrodes PO7/8 the most appropriate for analysing the symmetry response? We examined this with mass univariate analysis^48,49^ which tests differences between symmetry and random waves at all points in the first interval and in the retention interval. Figure 5 shows a statistical map similar in both tasks. Electrodes PO7 and PO8 show consistent differences in the second half of the visible interval and in the retention interval (because of grand-average reference this was coupled with a positive difference at frontal electrodes). This alternative visualization of the SPN confirms that (1) our a priori electrode choice was appropriate, (2) the SPN persisted, and (3) ERPs results were similar in both tasks.

### ERP response to the second pattern

We examined ERPs generated by the second pattern to test whether they were affected by the information preceding it. We computed amplitude in the 1700–2000 ms window, and baseline correct to 200 ms before the onset of the second pattern. We explored the response to the different sequence trials (right column of Fig. 2). Amplitude was analysed with two within-subjects factors [(Second Regularity (Symmetry, Asymmetry) X First regularity (Symmetry, Asymmetry)] and one between-subjects factor [Task (Shape, Colour)]. Amplitude was more negative for symmetrical patterns than asymmetrical patterns (F (1, 38) = 29.028, p < 0.001, partial η^2^ = 0.433). There were no other effects or interactions (F (1, 38) <3.256, p > 0.079).

**Figure 6:**
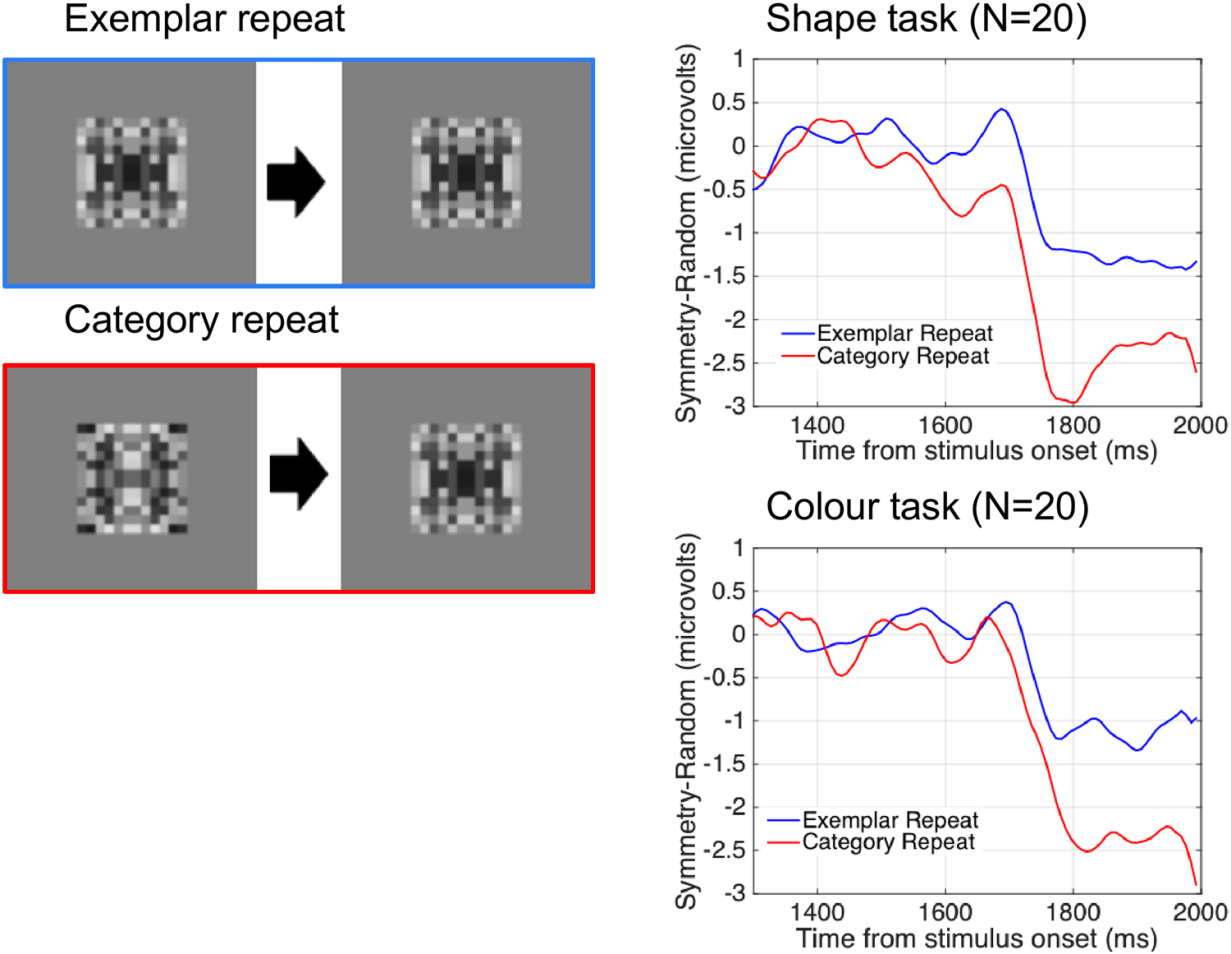
The SPN is shown as difference waves generated by the second pattern (relative to an asymmetrical pattern). Note that both first and second patterns are symmetrical. The second symmetry in a pair of two identical symmetries (Exemplar Repeat) generated a smaller (i.e. less negative) response than the second symmetry in a pair of different symmetries (Category Repeat).

It is interesting to compare SPNs generated by novel and by repeated stimuli. Figure 6 shows the SPN in the case of a pair of symmetries (pattern 1 and pattern 2). These pairs were either an Exemplar repeat, where the same pattern is shown twice, or a Category repeat, where one symmetrical pattern is followed by a different symmetrical pattern. The difference waves was computed by subtracting the corresponding asymmetry condition (i.e. identical asymmetry pattern or different asymmetry pattern). The neural response was larger for Category repeat than Exemplar repeat. This advantage was of approximately the same magnitude in both tasks, even though the two kinds of repeats are not equally frequent (see design in Fig. 2). It is unlikely that expectations played a role given the lack of a difference when observers performed two different tasks, only one of which required processing of shape information.

To analyse these effects, we used a mixed ANOVA. There were two within-subjects factors [Repeat Type (Exemplar, Category) X Regularity (Symmetry, Asymmetry)] and one between-subjects factor [Task (Shape, Colour)]. There was a main effect of Regularity (F (1, 38) = 29.657, p < 0.001, partial η^2^ = 0.438) and a Repeat Type X Regularity interaction (F (1, 38) = 5.268, p = 0.027, partial η^2^ = 0.122), resulting from a stronger effect of Regularity for Category repeat (t (39) = 4.742, p < 0.001) than for Exemplar repeat (t (39) = 3.652, p = 0.001). There were no effects involving the factor Task (F (1, 38) < 0.860, p > 0.960).

## Discussion

We discuss the results in terms of the three events in the sequence: Pattern 1, Blank interval, and Pattern 2. There was a posterior negativity generated by the first pattern and similar for both tasks. This replicated previous studies which show a task independent response to image symmetry (Fig. 1).

With respect to the blank interval, the SPN spanned the one-second interval between pattern 1 and pattern 2. The persistence of the SPN clarifies aspects of the SPN recorded in other studies. It has been observed that the SPN lasts for at least one-second when the stimulus remains visible (as shown in Fig. 1). Our results suggest that internal dynamics of the symmetry-sensitive network, not just stimulus duration, is responsible for the sustained nature of the SPN. As there was no luminance mask, some sustained activity was expected. It could be argued that the persistent SPN during the blank interval is merely the sluggish decay of the initial symmetry response. For example, Niimi, Watanabe and Yokosawa^50^ have shown how persistence contribute to perception of symmetry. However, the neural response after offset had not been tested before. Here we report activity over a 1 second blank interval, and no modulation by task (Shape matching or Colour). This shows that attending to information in the shape discrimination task did not enhance this sustained activity, even after stimulus offset.

In our study a blank screen was used in the retention interval. Supplementary experiments show that the SPN is not visible when luminance masks are used (Supplementary Experiment 1S and 2S), and that it is possible for participants to perform a same/different task even when the SPN is not sustained. However, a visually-driven SPN seems to be a pre-requisite for accurate performance (Supplementary Experiment 2S).

The SPN is similar to other components in terms of latency and topography, and this requires carefully consideration. For instance, the name SPN is similar to the sustained posterior contralateral negativity (SPCN). In a study by Jolicoeur, Brisson and Robitaille^51^ characters appeared in either the left or right visual hemifields for 100 ms, and a cue indicated which characters had to be memorised. SPCN amplitude scaled with memory load after stimulus offset (unlike the N2pc, which indexed direction of spatial attention, but not memory load). The SPCN is computed as the difference between ipsilateral and contralateral electrodes. In contrast, the SPN is the difference between regular and random conditions at bilateral posterior electrodes. A persistent SPN may reflect maintenance of the symmetry representations in the extrastriate cortex, while the SPCN may reflect visual representations in either left or right locations. These are not the same components (see also Wright *et al*.^32,52^). As stated by Jolicoeur *et al*.: “The double subtraction used to compute the SPCN removes any effect that does not depend on the position of the stimuli” (p 169).

Wright *et al*.^32^ discuss the possibility that spatial attention plays a part in symmetry tasks. In their Experiment 2, patterns were present on one side and there was nothing on the other side. Here participants would presumably shift spatial attention to the pattern, be it reflection or random. This should produce a similar SPCN. Yet, amplitude was more negative for reflection than random. Another reason why it is unlikely that the SPN for central stimuli is an attentional component is that the SPN is similar when patterns are presented with horizontal or vertical axis^52^, even though axis orientation alters the distribution of spatial attention. Moreover, SPN amplitude can be predicted by models that quantify perceptual goodness^23^. It would be difficult to explain such precise SPN results by differences in the deployment of attention. We conclude that although there is some overlap between attention-related ERPs and the SPN in terms of latency and topography, these ERP are generated by different mechanisms.

The SPN is related to the late component found when contrasting recognizable objects with scrambled controls^53,54^. These are neural responses to objects or perceptual wholes, and this may be related to the response to symmetry given that symmetry is related to objectness^40,55^. However, as the SPN is measured as a differential response to symmetrical and asymmetrical wholes, the SPN is a specific index of the symmetry response. Here we show that this response persists after stimulus offset.

Cognitive science historically makes a sharp distinction between perception and memory. However, modern syntheses suggest a near-total overlap^56–59^. Visual systems are optimized for certain modes of representation, and working memory probably involves persistence of these representations after stimulus offset. Fuster^58^ described this proposal as the emergence of a new paradigm. The persistent SPN we recorded is consistent with this approach. However, more work is required to interpret the persistent SPN as a neural signature of symmetry in memory^60^. One would like to see coupling between persistent SPN activity and behavioural performance. This has not been conclusively established, although Kohler, Cottereau and Norcia^61^ reported evidence of judgment-related activity in the extrastriate network. A novel experiment on the link between SPN and performance is reported in Supplementary Materials. It shows that on trials in which participants made the correct judgement, the symmetry wave was more negative than the random wave (SPN). However, on trials in which participants made an incorrect judgement there was no difference.

We now turn to the discussion of the response to the second pattern. This response to symmetry was present, as expected, and enhanced when symmetry was also present in the first interval. This reflect what we have already seen, namely that the sustained negativity was still present during the blank interval.

When the first and second patterns were symmetrical, in some cases they were identical patterns, and in other cases they were different patterns (same category but different exemplars). Similarly, when the first and second patterns were asymmetrical, in some cases they were identical, and in other cases they were different. The SPN was significantly larger in the category repeat condition (when two different examples were shown) than in the exemplar repeat condition (when two identical examples were shown).

Prima facie this appears paradoxical. If the sustained activity is a response to the image presented in the first interval, one could expect greater summation when the patterns are identical. This would be the case if summation is due to shared spatial properties and identical retinal positions of the elements. On the other hand, different exemplars share regularity at a higher level, as identical Gestalts. Therefore, the second pattern adds consistent regularity information to the original Gestalt. This can be understood as a form of symmetry priming, rather than image priming.

These results show that symmetry representations are maintained, and they have a systematic effect on subsequent symmetry processing. As discussed in the introduction, there is behavioural evidence that different symmetrical patterns can prime response to the second exemplar. Specifically, Sharman and Gheorghiu^47^ anticipated this effect in light of their psychophysical results. We conclude that the onset of new correspondences around already encoded axes boosts the extrastriate symmetry response.

It is also interesting that the category repeat advantage was of comparable magnitude in both tasks. In contrast, there was a left parietal ERP difference between same and different trials in the Shape task, but not in the Colour Task (see Supplementary Materials).

In summary, the extrastriate symmetry response was task independent, and remained so for one second after the symmetrical patterns disappear. This kind of symmetry activation (persistent activation after stimulus offset) was found even when observers were not performing a task in which shape information was relevant. Interestingly, we found that processing different symmetries, interleaved by short temporal intervals, boosts the regularity-specific extrastriate activity. Probably, encoding axis of symmetry primes response to new regular local correspondences. This priming effect needs to be further explored in future research.

## Method

### Participants

Twenty individuals participated in the Shape matching task (aged 18 to 33, 1 left handed, 8 male). Another 20 participated in the Colour task (aged 18 to 33, 2 left handed, 3 male). Participants had normal or corrected-to-normal vision. The study was approved by the Health and Life Sciences Committee on Research Ethics (Psychology, Health and Society) at the University of Liverpool and conducted in accordance with the Declaration of Helsinki (revised 2008). All participants signed an informed consent form.

### Apparatus

Participants sat in a dark, electrically shielded room, 100 cm from a 30 × 40 cm CRT monitor with 60 Hz refresh rate. Distance from the monitor was controlled by the use of a chinrest. EEG data were recorded continuously from 64 scalp electrodes arranged by the international 10–20 system. We used the BioSemi active-two system EEG system, sampling at 512 Hz, with 0.16 to 100 Hz bandpass filter. The images and presentation were programmed in Python using PsychoPy libraries^62^.

### Stimuli

Symmetrical and asymmetrical square field patterns were generated on each trial, and therefore no trial ever presented images used before. All symmetrical patterns were two-fold reflections, similar to those used in previous studies^30,63^. Two-fold reflection generates a large SPN compared to single-axis reflection and other symmetry types^23^. Each pattern was made of 12 × 12 grey squares. Each side was approximately 0.36° of visual angle, and the patterns were approximately 4.32° × 4.32°. The luminance of each square was chosen at random between −0.8 to +0.8 in PsychoPy RGB coordinates, giving luminance values from 0.15 cd/m^2^ to 50.2 cd/m^2^ on our CRT monitor. Background grey was set at 7.3 cd/m^2^.

### Procedure (Shape matching task)

Each of the eight conditions shown in Fig. 2 were repeated 20 times with different, novel exemplars. In 80 trials a symmetrical pattern was presented first, and in 80 trials an asymmetrical pattern was presented first. For 80 trials the correct response was Same, and for 80 trials the correct response was Different. To balance the design, 75% of trials with a symmetrical pattern were followed by a symmetrical pattern, and 25% of trials had a category switch from symmetry to asymmetry or vice versa (note the different combinations illustrated in Fig. 2). The buttons used to report Same and Different were counterbalanced across subjects.

The same-different dimension was orthogonal to regularity (Fig. 2). There were two kinds of Same trial (identical symmetries or identical asymmetry patterns), but four kinds of Different trial (two different symmetries, two different asymmetry patterns, symmetry followed by asymmetry, or asymmetry followed by symmetry). This design ensured that participants could not perform the Shape matching task by merely assigning a verbal label to the first pattern (such as symmetry) and remembering the label alone. Instead, it encouraged them to retain information during the blank interval. This design maximises task differences that might modulate the post stimulus SPN.

### Procedure (Colour task)

The conditions in the Colour task were identical to the Shape matching task, except that an *additional* 40 coloured trials were included (25% of total trials). In these trials both pattern 1 and pattern 2 were blue (Fig. 3). The additional coloured trials were not included in the ERP analysis. By excluding these trials, the between-subjects factor Task was balanced, so the same trial types were equally represented in both Shape matching and Colour tasks.

### Data analysis

#### Grand-average ERP analysis

Data were analysed offline using the eeglab 13.4.4b toolbox^64^ in Matlab 2014b. Pre-processing was designed to be as similar as possible to previous EEG studies with visual symmetry^22^. EEG data were re-referenced to a scalp average, and downsampled to 128 Hz. We then segmented the data into −1 to 2 second epochs. Independent Components Analysis (ICA) was used to remove oculomotor and other artefacts^65^. In the Shape matching task, ICA components were removed manually (mean = 8.6, min 2, max = 17) and mean trial exclusion rate was 20%. In the Colour task an average of 9.55 ICA components were removed from each participant (min = 3, max = 17). After ICA, trials where amplitude exceeded +/− 100 μV at any electrode were excluded. Mean trial exclusion rate was 22%.

The PO7 and PO8 electrodes were chosen *a priori* for ERP analysis. The SPN at these electrodes has been consistently associated with the extrastriate symmetry response, and we are interested in persistent activation of the symmetry network (rather than persistent activity elsewhere in the brain). Although some previous SPN studies used larger electrode clusters to examine differences between hemispheres^32^ PO7/8 are often used when symmetry is presented in the centre of the visual field^23^.

#### Mass Univariate Analysis

Mass univariate analysis was used to assess the spatiotemporal development of the symmetry response. The analysis was conducted by using the LIMO EEG toolbox^49,66^. This analysis deals both with within-subject variance (i.e., single trial analyses) and between-subject variance; data are analysed using a hierarchical general linear model where parameters are estimated for each subject at each time point and each electrode independently (1st level analyses). Estimated parameters from the first level analyses are then integrated across subjects (2nd level analysis). With this approach, we conducted a paired t-test (categorical variable: symmetry vs. asymmetry). Figure 5 plots the t-tests at all timepoints and electrodes (collapsed across the three experiments). The two panels show results without correction for multiple comparisons. A criterion p value of 0.05 was used, so all areas in grey correspond to p > 0.05.

## Supplementary information

Supplementary material
