# Supplementary material for: Sustained response to symmetry in extrastriate areas after stimulus offset: An EEG study
Source: Sci Rep. 2019 Mar 13;9:4401. doi: 10.1038/s41598-019-40580-z (PMC6416322; doi:10.1038/s41598-019-40580-z)
Supplement: Supplementary file 1 — Supplementary material [file 41598_2019_40580_MOESM1_ESM.docx]

**Sustained response to symmetry in extrastriate areas after stimulus offset:**

**An EEG study**

Marco Bertamini^1*^, Giulia Rampone^2^, Jennifer Oulton^3^, Semir Tatlidil^1^, and Alexis D.J. Makin^1^

^1^ University of Liverpool, Department of Psychological Science, Liverpool, L697ZA, UK

^2^ University of Liverpool, School of Psychology, Liverpool, L697ZA, UK

^3^ Liverpool John Moores University, Sport and Exercise Sciences, Liverpool, L2 2QP, UK

* m.bertamini@liv.ac.uk

**Supplementary Materials –**

**Additional analysis of the response to pattern 2 (Experiment 1)**

We analysed ERPs generated by the second pattern collapsing over the Symmetry-Asymmetry factor, and excluding oddball trials. There were an equal number of Same and Different trials. A difference was evident when comparing topographic plots from same trials (left columns main manuscript Figure 3) to different trials (right columns of main manuscript Figure 3). In the Same/Different task, there was a left parietal effect, where amplitude was more negative from around 1750 to 2000 ms when the same pattern was repeated than when a new pattern was presented (Figure 1S left column). This left parietal effect was absent in the Oddball Detection task (Figure 1S right column). These results are generally consistent with the ERP memory literature, where left parietal old/new effects have been documented ^e^^.g. 1^.

To analyse this left parietal effect, we chose a cluster of electrodes shown in Figure 1S (PO3, PO7, P3, P5 and P7). This impression was confirmed with a mixed ANOVA with one within-subjects factor [Second Pattern (Same, Different)] and one between-subjects factor [Task (Same/Different vs. Oddball Detection)]. There was a main effect of Second pattern (F (1,38) = 9.841, p = 0.003, partial η^2^ = 0.206), and a Second Pattern X Task interaction (F (1,38) = 5.839, p = 0.021, partial η^2^ = 0.133). There was a strong left-sided effect of Second Pattern in the Same/Different task (t (19) = 4.212, p < 0.001), but not in the Oddball Detection task (t (19) = 0.479, p = 0.637).

This interaction suggests that the matching or mismatching relationship between the first and second patterns was more salient in Same/Different task than in the Oddball Detection task. This is not surprising, given the different cognitive demands of the tasks. This left parietal effect contrasts with the persistent SPN activity during the retention interval, and the difference between exemplar and category repeats. Both these effects were task independent.

**
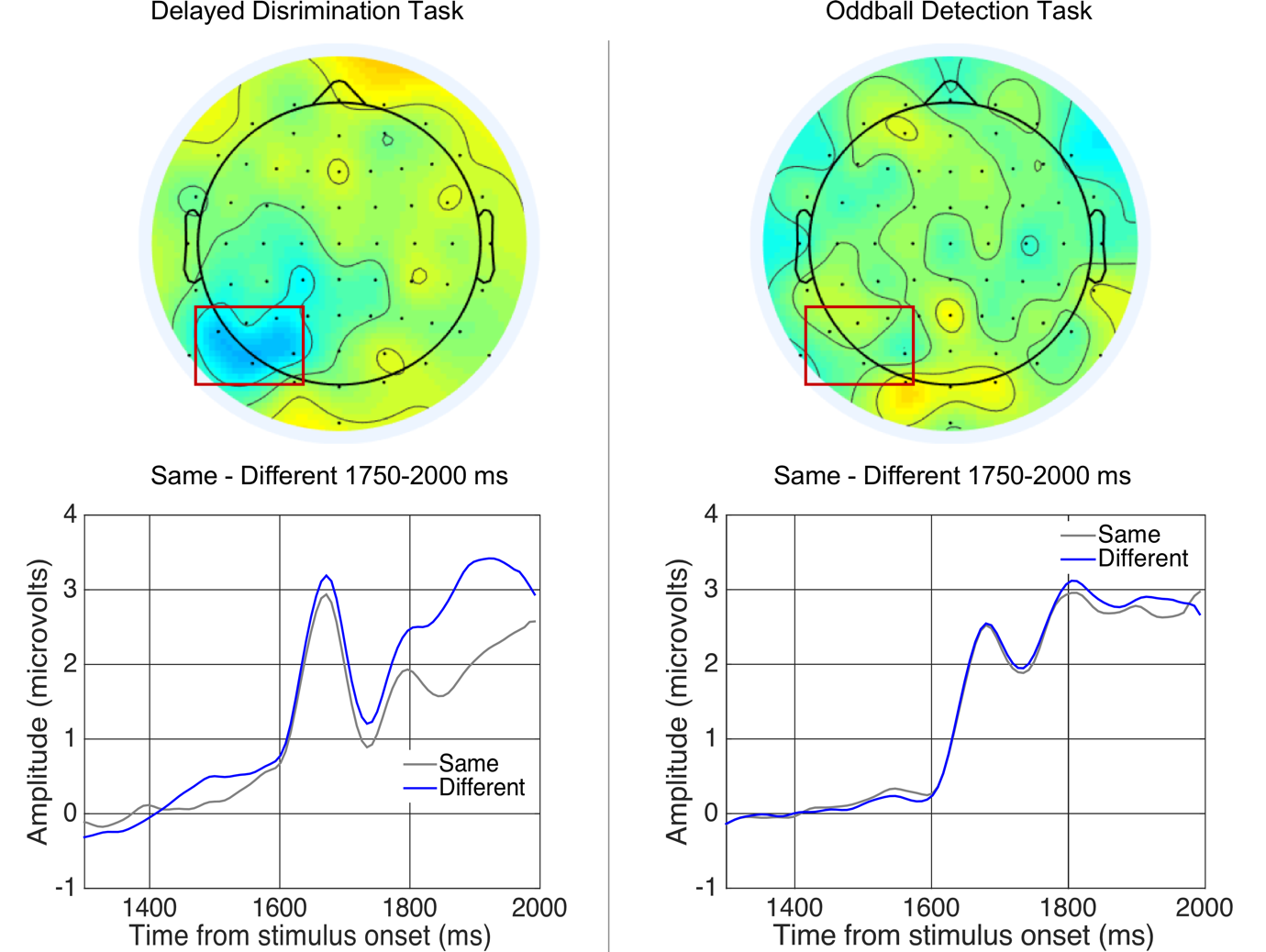
Figure 1S.** Grand Average ERPs in response to the second pattern in each trial. Left column shows results from the Same/Different task, the right column shows results from the Oddball Detection task. The red rectangle captures the left posterior electrodes used in the ERP plots below.

**Supplementary Experiments**

We report two additional experiments with visual noise masking. These studies show that the post stimulus SPN can be reduced or abolished by visual noise masking.

**Experiment 1S**

Supplementary Experiment 1S explored the persistence of extrastriate symmetry activation when patterns are masked. In half the trials, we presented abstract patterns with two-fold reflectional symmetry. In the other half of the trials, we presented asymmetry (Figure 2S.A). Stimuli were presented for either 150, 500 or 1000 ms, then replaced with a random mask. The duration of the mask was inversely related to the duration of the pattern, so that trial duration was always 2000 ms (Figure 2S.B). We were particularly interested in posterior ERPs generated during the mask interval.

To anticipate the results, we found that mask onset made the SPN undetectable. In medium and long duration conditions, some persistent symmetry-related activity re-emerged, even though the stimulus on the screen was asymmetrical at this point. However, the amplitude of this post-mask response was low compared to the pre- mask visually driven SPN.

**Supplementary Experiment 1S Materials and Methods**

**Participants and apparatus**

Thirty-six participants were involved in Experiment 1S (10 males, 5 left-handed, aged 18-49, mean age = 21). The apparatus and stimuli were the same as the main experiment.

**Procedure**

There were 6 conditions [2 Regularity (Symmetry, Random) X 3 Pre-Mask Duration (150, 500 or 1000 ms)]. Each condition was repeated 72 times, giving 432 experimental trials in total. Trials were presented in a different randomized order for each participant.

Figure 2S shows the sequence of events on an individual trial. Participants were instructed to maintain central fixation for the whole epoch. After this, participants entered their response by pressing buttons labelled ‘Reflection’ or ‘Random’. The response key mapping alternated unpredictably on each trial. The experimenter explained that the task was to discriminate symmetry from random *in the pre-mask interval*, and that the masks would never contain symmetry. There was a 24-trial practice block in which participants could familiarize themselves with the task.

**
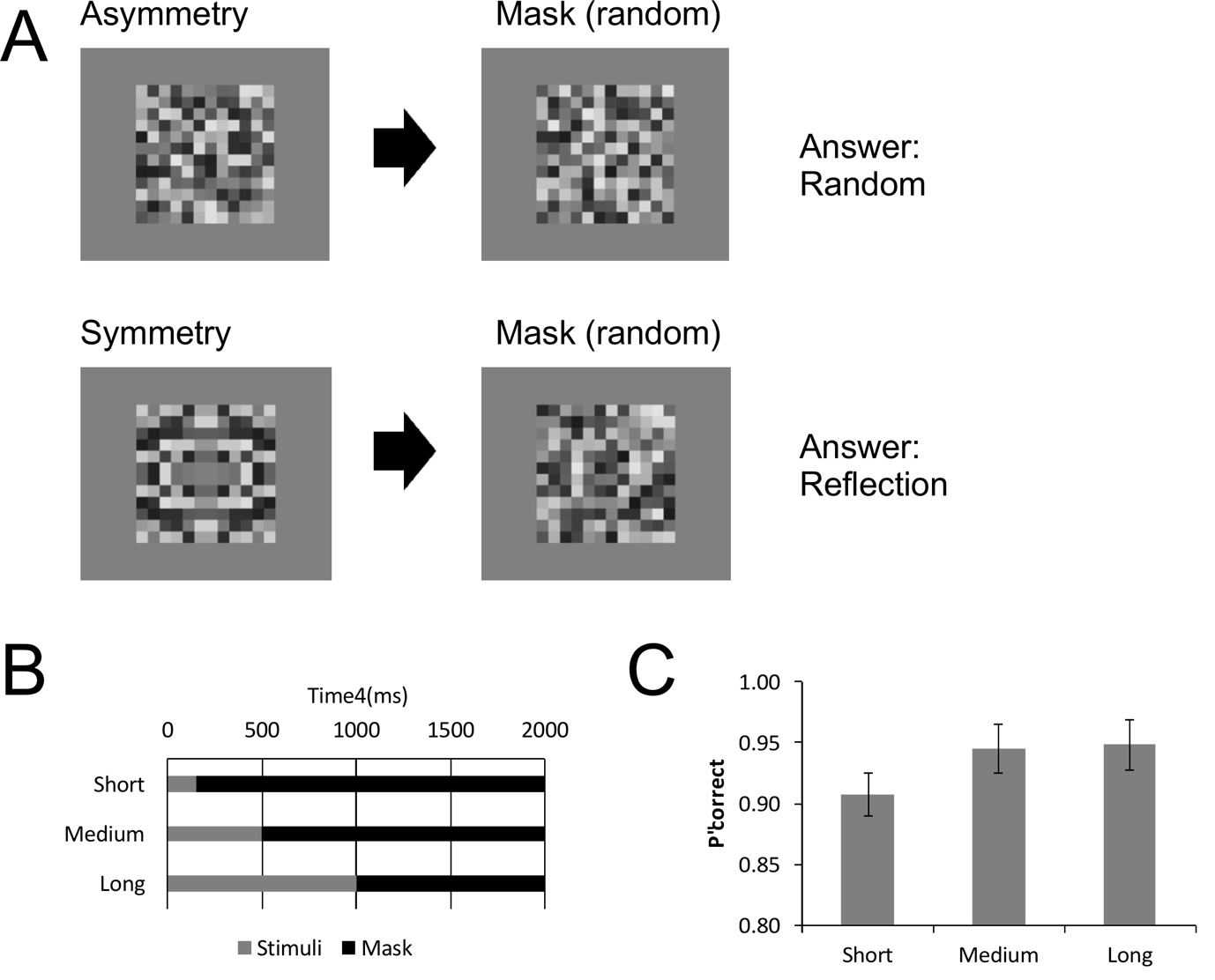
Figure 2S. Supplementary Experiment 1S Method**. A) Two trial types: a two-fold reflectional symmetry replaced with a mask, or a random pattern replaced with a mask. These are examples, in the experiment every trial used a novel set of patterns. B) Schematic showing the structure of the short, long and medium trials. C) Behavioural performance in the three conditions. Error bars = +/- 1 S.E.M.

**Data analysis**

Data analysis conventions were identical to the main experiment. On average, 10 ICA components were removed from each participant (min = 2, max = 22). After ICA, trials where amplitude exceeded +/- 100 μV at any electrode were excluded. Approximately the same number of trials were excluded from each condition (mean exclusion rate ranged from 11% to 13%). Trial exclusion rate varied between participants, but no more than 50% of trials were excluded from any condition (min = 0%, max = 48.61%).

**Supplementary Experiment 1S Results**

**Behavioural results**

Participants correctly discriminated the pre-mask pattern as either symmetry or random on most trials (Figure 2C). Mean performance was 91% correct in the short condition, where the pre-mask pattern was presented for 150 ms. However, there was a small but significant improvement in the medium condition, where patterns were presented for 500 ms (94% correct) and long condition, where patterns were presented for 1000 ms (95% correct), (χ^2^(2) =24.365, p<0.001). A minority of trials where participants entered incorrect judgements were still included in ERP analysis (exclusion of the error trials did not change the shape of the ERPs or statistical analysis).

**ERP results**

Grand average ERPs from PO7 and PO8 electrodes are shown in Figure 3S. The SPN as a difference wave can be seen in Figure 4S. As expected, an SPN was generated by symmetry. That is, amplitude was lower for symmetrical than asymmetrical trials when patterns were on the screen (purple boxes). Although the SPN was temporarily disrupted by mask onset (green boxes), it re-emerged in the medium and long conditions (red boxes). This component could reflect persistence of the SPN activity.

**
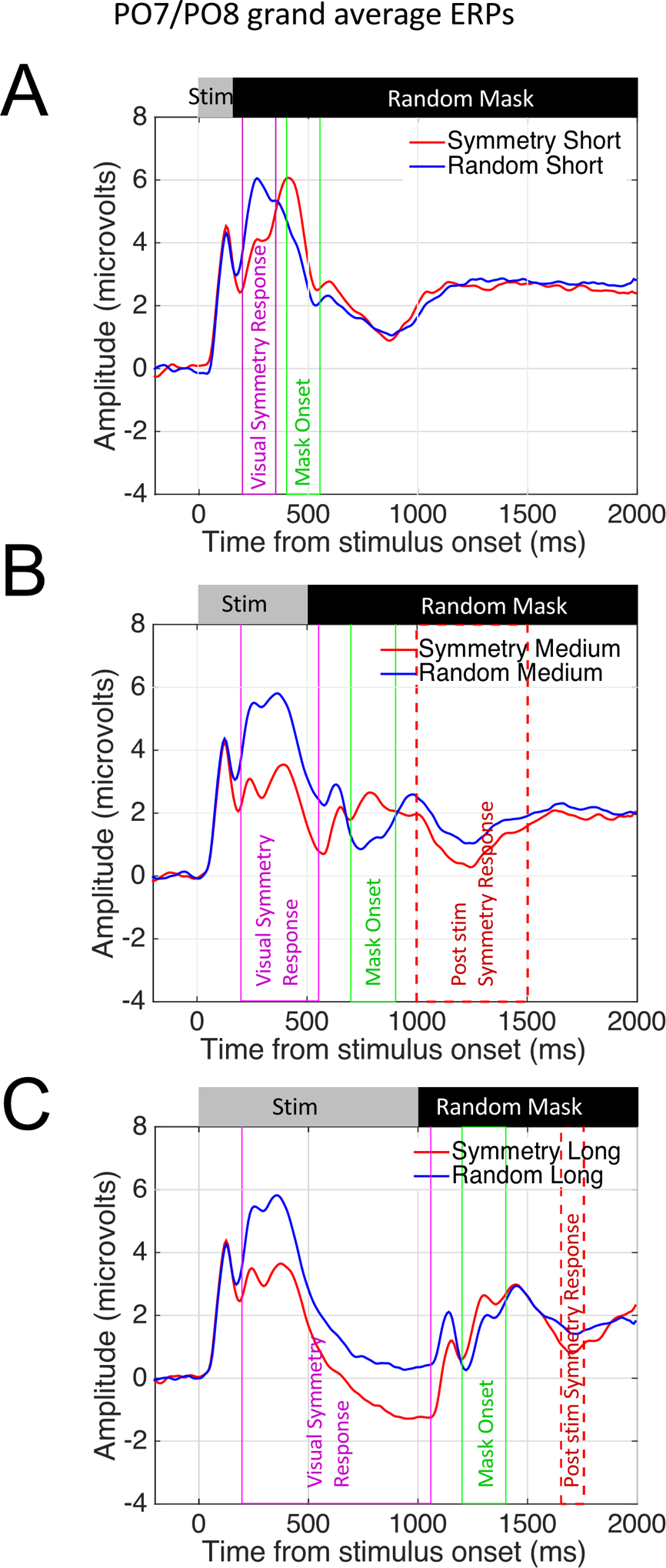
**

**Figure 3S: Experiment 1S results.** A) Grand average ERPs from electrodes PO7/8 in short, medium and long conditions. There are two intervals where the symmetrical and random ERPs diverge: shortly after the onset of the first pattern (SPN, purple box), and during the mask interval. Amplitude was more negative following a symmetrical than a random pre-mask pattern (red box). The post-mask SPN was only evident in the medium and long conditions.

**
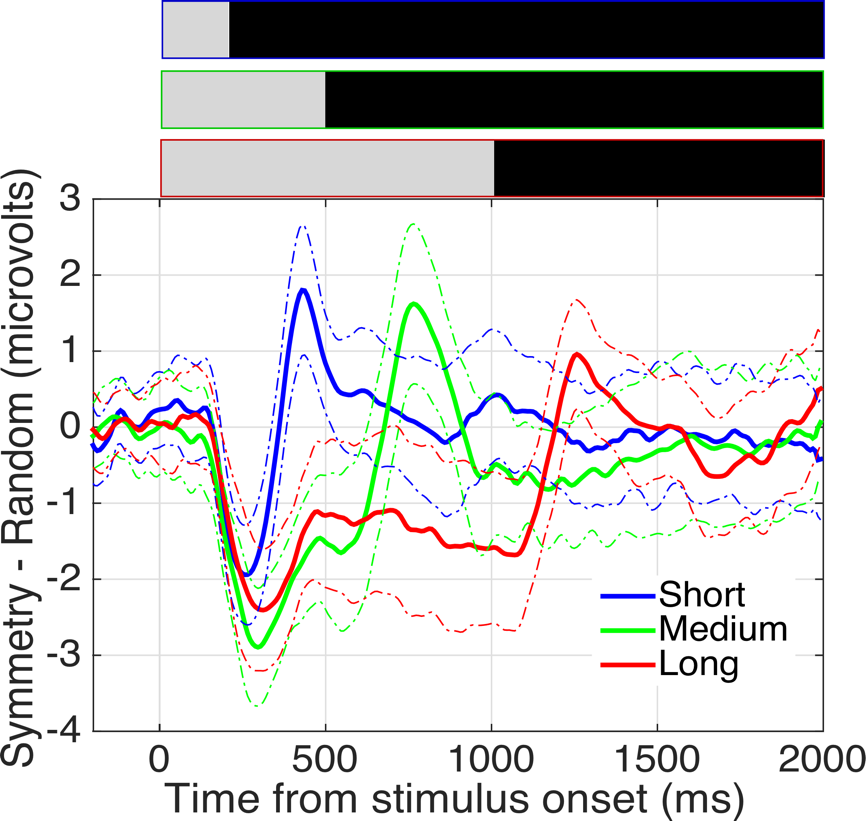
**

**Figure 4S**. Grand average difference waves from electrodes PO7/8 in short, medium and long conditions. Bars above the plot indicate pattern and mask intervals in each duration conditions (colour coded). Dashed lines show 95% CI.

We analysed the visually-driven SPN in the short (200-350 ms), medium (200-550 ms) and long time-windows (200-1050 ms). It was necessary to use different time windows because the onset of the mask terminated the visual symmetry response at different time points in each condition. A 2 X 3 Repeated measures ANOVA [2 Regularity (Symmetry, Random) X 3 Pre-Mask Duration (150, 500 or 1000 ms)] found main effects of Regularity (F (1,70) = 72.221, p < 0.001, partial η^2^ = 0.674) and of Pre-Mask Duration (F (1.597, 55.903) = 66.916, p < 0.001, partial η^2^ = 0.657) but no interaction (F (2,70) = 2.578, p = 0.083).

To analyse the post mask SPN, we chose different windows of 1000-1500 in the medium condition and 1650-1750 in the long condition. A 2 X 2 Repeated measures ANOVA [Regularity (Symmetry, Random) X Pre-Mask Duration] found a main effect of Regularity (F (1, 35) = 8.915, p = 0.005, partial η^2^ = 0.203). There was no main effect of Pre-Mask Duration (F (1,35) = 0.255, p = 0.617) and no interaction (F (1,35) = 0.003, p = 0.959).

Additional analysis of the medium and long duration conditions revealed that the visually driven SPN was significantly stronger than the post-stimulus SPN (-1.82 vs. - 0.60 μV, t (35) = 4.265, p < 0.001).

**Supplementary Experiment 1S Discussion.**

Experiment 1S demonstrates that a visual mask terminates the SPN, whether it appeared at 250, 500 or 1000 ms post stimulus. There was, however, a persistent but weak SPN activity post-mask in the medium and long conditions. There was no persistent SPN activity in the short condition, even though participants could perform the task successfully on most trials.

The onset of the ask caused a temporary positive peak in the difference wave (Figure 4S). It is likely that the onset of a random pattern after a symmetrical pattern (symmetry > noise) was more visually disruptive than the onset of a random pattern after another random pattern (noise > noise). These onset / offset asymmetry dynamics are well documented in the SSVEP literature ^2^.

The most parsimonious way of interpreting the results is to say that the SPN persisted weakly after the mask, but was temporarily disrupted by the visual transient caused by mask onset. However, this persistent SPN activity was reduced compared to the visually driven SPN. This is different from the main experiment, where the SPN continued uninterrupted post stimulus. Noise masks strongly disrupt persistent symmetry representations or create a signal that makes recording of the SPN impossible.

**Supplementary Experiment 2S**

The aim of Experiment 2S was to replicate the main Same/Different task with a few manipulations to abolish ceiling effects, and thus ascertain the relationship between persistent SPN activity and performance. The duration of pattern 1 was *reduced* by 50% to just 250 ms, and the retention interval was *extended* by 50%, to 1500 ms. A mask was presented in the interval (this was a slightly larger square than the patterns 1 and 2, so it could not be mistaken with the comparison patterns).

The four trial types are shown in Figure 5S [Regularity (Symmetry, Random) X Trial type (Same, Different)]. An interesting comparison is between trials where participants made a *correct judgement* (i.e. Same on a Same trial, or Different on a Different trial) and trials where they made an *incorrect judgement* (i.e. Same on a Different trial or Different on a Same trial). However, we were not interested in trials where participants merely guessed the correct answer. To overcome this, participants were given a ‘Do Not Know’ option, and such responses were classed as *incorrect* for ERP analysis.

To encourage task engagement, we added a game-like element, where participants won one point for getting the correct answer and lost one point for giving the incorrect answer (Figure 6S). The Do Not Know option did not result in any losses or gains. The number of points accumulated was displayed between blocks as a running total.

Pattern 1 and 2 had the same regularity (both either symmetrical or random). This feature *discouraged* participants from attending to category type alone, and *encouraged* them to attend to the exact arrangement of grey-scale squares.

**
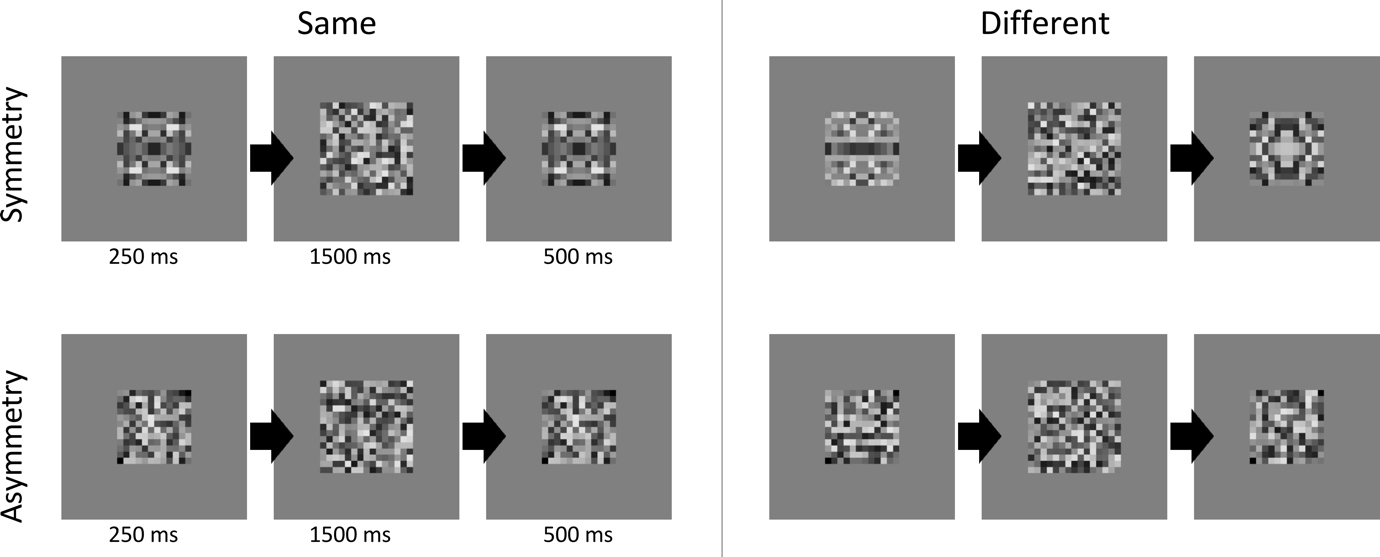
Figure 5S.** The four trial types in Supplementary Experiment 2S.

**Supplementary Experiment 2S Materials and Methods**

A sample of 20 participants were involved in Experiment 2S (age 19-51, mean age = 25, 3 left handed, 7 male). These individuals did not take part in Experiment 1S. The stimuli were the same as Experiment 1S, however the central mask was 15 X 15 square (5.4 X 5.4 degrees), rather than 12X12 (4.32 X 4.32 degrees). The four trial types are shown in Figure 6S. There were 80 repeats of each type, giving 320 trials in total.


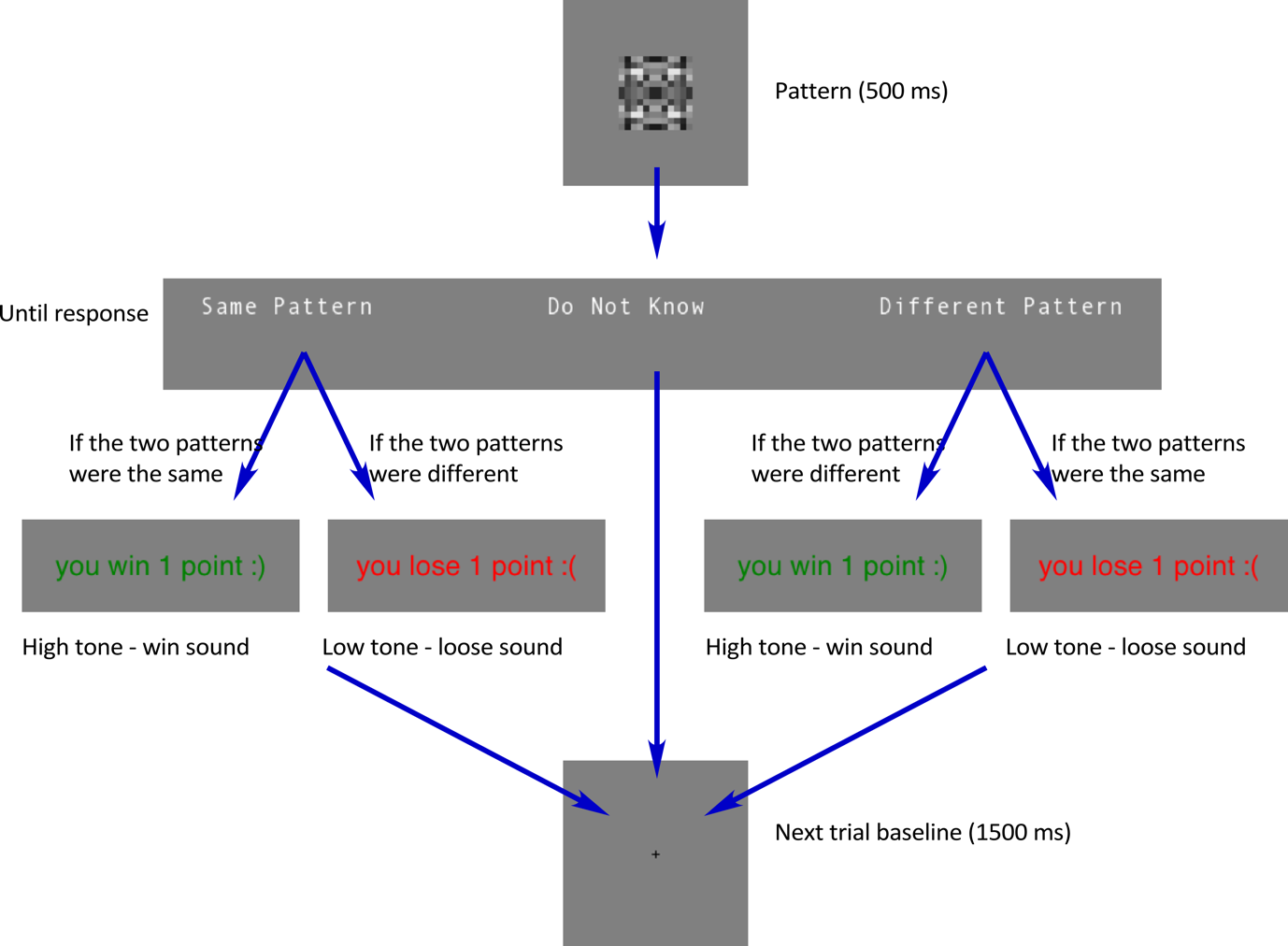


**Figure 6S.** Trial structure in Supplementary Experiment 2S.

On average, 9.85 ICA components were removed from each participant (min 2, max = 16) and mean trial exclusion rate was around 17.5% in all conditions. ERPs were analysed with a 2X2 Repeated measures ANOVA [Judgement (Correct, Incorrect) X Regularity (Symmetry, Random)]. Unlike previous analyses, the average ERPs entered were not based on the approximately same number of trials. There were more trials where participants made correct judgement (81.6%) than incorrect judgement (18.4%). The average (and minimum) number of included trials were as follows: Symmetry Correct = 117.25 trials (min = 66), Random Correct = 100.8, (min = 37) Symmetry Incorrect = 20.15 (min = 7) Random Incorrect = 25.75 (min = 7).

**Supplementary Experiment 2S results**

**Behavioural results**

Figure 7S.A shows that participants made the correct judgment on most trials (81.6% vs. 18.4%). Interestingly, they were more likely to be correct in the Symmetry condition than that the Asymmetry condition (Z = 3.921, p < 0.001) but more likely to enter a Do Not Know response in the random condition (Z = 3.920, p < 0.001). Clearly symmetrical patterns were easier to encode and compare with subsequent patterns, possibly because 75% of the visual information was redundant ^3^^,4^. This is consistent with previous findings ^5^^,6^.

**ERP results**

ERPs at PO7/8 electrodes are shown in Figure 7S.B-F. The visually driven SPN was only present on Correct trials. Figure 7S.E zooms in on the first 400 ms to highlight this effect. We can see that the waves on correct symmetry trials were different from all others.

The SPN was measured from 200-350 ms. Impressions were confirmed in a 2X2 repeated measures ANOVA [Judgement (Correct, Incorrect) X Regularity (Symmetry, Random)]. There were main effects of Judgment (F (1,19) = 9.665, p = 0.006, partial η^2^ = 0.337), Regularity (F (1,19) = 7.610, p = 0.012, partial η^2^ = 0.286) and a Judgement X Regularity interaction (F (1,19) = 6.922, p = 0.016, partial η^2^ = 0.267). On trials when participants made the correct judgement, the symmetry wave was more negative than the random wave (t (19) = -5.535, p < 0.001). Conversely, on trials where participants made an incorrect judgement there was no difference (t (19) = 0.021, p = 0.983). The symmetry wave on *incorrect trials* did not differ from random wave on *correct trials* (t (19) = -0.482, p = 0.635).

These SPN effects could have trivial explanations. For instance, participants might simply shut their eyes on some trials, thereby eliminating the SPN and reducing performance to chance levels. However, we note that the P1 component was similar on correct and incorrect trials, so this class of explanation is less plausible. Unsmoothed ERPs give a true representation of P1 peak amplitude in Figure 7S.E. P1 amplitude was measured at 130-150 ms and 2X2 repeated measures ANOVA [Judgement (Correct, Incorrect) X Regularity (Symmetry, Random)] found no significant effects (Borderline Regularity X Judgment interaction only (F (1,19) = 3.355, p = 0.083). The slight difference at N1 was continuous with the SPN, so we do not analyse the N1 component separately.

There was no persistent SPN during the masked retention interval, either on correct or incorrect trials (Figure 7S.C). In fact, amplitude was *more positive* after a symmetrical pattern 1, and this was more pronounced on *incorrect trials*. For consistency with the main experiment, we chose a window from 200 ms into the interval until 50 ms into pattern 2 (450 to 1800 ms). There was a main effect of Regularity (F (1,19) = 6.942, p = 0.016, partial η^2^ = 0.268), but no effect of Judgement (F (1,19) = 0.004, p = 0.948) or Regularity X Judgment interaction (F (1,19) = 0.871, p = 0.362).


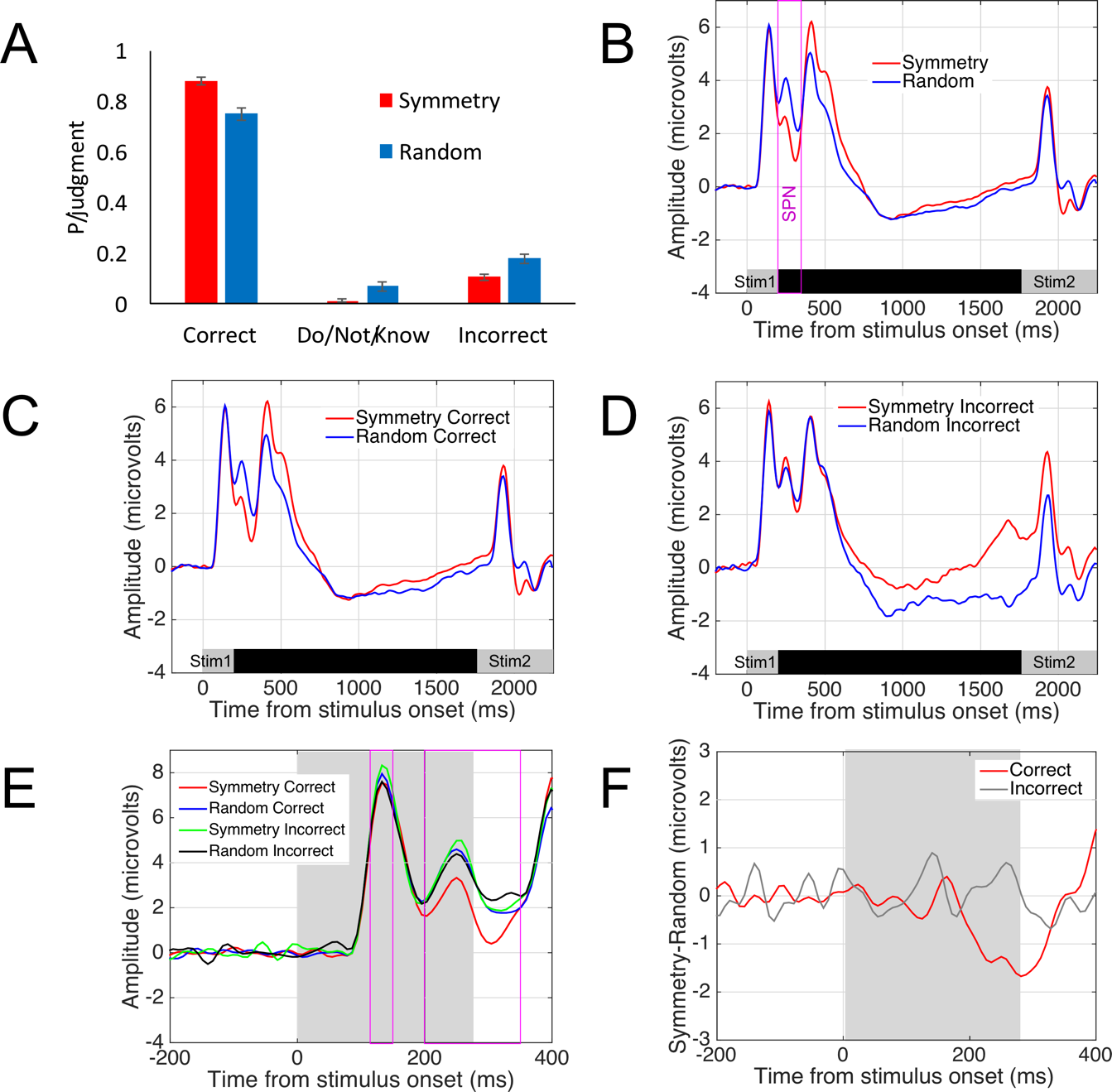


**Figure 7S. Experiment 2S Results.** A) proportion of ‘Correct’, ‘Do Not Know’ and ‘Incorrect’ judgements on the symmetry and random trials. B) Grand-Average ERPs in PO7/8 electrodes. There was a short SPN just after the onset of pattern 1, but no persistent SPN during the interval. C and D show ERPs in correct and incorrect trials separately. E) and F) zoom in the first 400 ms of the trial to highlight relationship between SPN and performance (Here ERPs are not smoothed so the P1 peak is enhanced compared to the plots above).

**Supplementary Experiment 2S discussion**

Experiment 2S revealed two interesting effects. First, there was no persistent SPN across the interval, even on trials where participants made the correct judgment. This shows that visual noise mask terminates the SPN after stimulus offset. It also shows that the post stimulus SPN is not essential for performance in these Same/Different tasks.

Second, we found that the visually driven SPN was only present on correct trials. It could be that the efficacy of the extrastriate network fluctuates on a trial-by-trial basis. There was a minority of trials where the retinal symmetry was not automatically detected. However, earlier visual responses, indexed by P1, were independent of judgement, and P1 amplitude was the same on correct and incorrect trials.

This dissociation between basic visual filtering (indexed by P1) and gestalt formation (indexed by SPN) is an interesting topic for future work. Process models of symmetry perception make this distinction between ubiquitous image filtering and secondary symmetry extraction ^7^^–9^. Meanwhile the holographic model (which predicts SPN amplitude well) quantifies the strength of post-filter representations ^3^^,4^. It thus seems reasonable to use P1 and SPN as tools to measure different states of the symmetry processing hierarchy in future research.

**References**

1. Rugg, M. D. & Curran, T. Event-related potentials and recognition memory. *Trends in Cognitive Sciences* **11,** 251–257 (2007).

2. Norcia, A. M., Appelbaum, L. G., Ales, J. M., Cottereau, B. R. & Rossion, B. The steady-state visual evoked potential in vision research: A review. *J. Vis.* **15,** 4 (2015).

3. van der Helm, P. A. & Leeuwenberg, E. L. J. Goodness of visual regularities: A nontransformational approach. *Psychol. Rev.* **103,** 429–456 (1996).

4. Makin, A. D. J. *et al.* An electrophysiological index of perceptual goodness. *Cereb. Cortex* **26,** 4416–4434 (2016).

5. Fox, J. The use of structural diagnostics in recognition. *J. Exp. Psychol. Hum. Percept. Perform.* **1,** 57–67 (1975).

6. Leeuwenberg, E. & Van Lier, R. Symmetry cues for matching mirrored objects. *Spat. Vis.* **18,** 1–23 (2005).

7. Dakin, S. C. & Herbert, A. M. The spatial region of integration for visual symmetry detection. *Proceedings. Biol. Sci.* **265,** 659–64 (1998).

8. Dakin, S. C. & Hess, R. F. The spatial mechanisms mediating symmetry perception. *Vision Res.* **37,** 2915–2930 (1997).

9. Rainville, S. J. M. & Kingdom, F. A. A. The functional role of oriented spatial filters in the perception of mirror symmetry — psychophysics and modeling. *Vision Res.* **40,** 2621–2644 (2000).
